# Twenty Thousand-Year-Old Huts at a Hunter-Gatherer Settlement in Eastern Jordan

**DOI:** 10.1371/journal.pone.0031447

**Published:** 2012-02-15

**Authors:** Lisa A. Maher, Tobias Richter, Danielle Macdonald, Matthew D. Jones, Louise Martin, Jay T. Stock

**Affiliations:** 1 Department of Anthropology, University of California, Berkeley, California, United States of America; 2 Department of Regional and Cross-Cultural Studies, University of Copenhagen, Copenhagen, Denmark; 3 Department of Anthropology, University of Toronto, Toronto, Ontario, Canada; 4 Department of Geography, University of Nottingham, Nottingham, United Kingdom; 5 Institute of Archaeology, University College London, London, United Kingdom; 6 Division of Biological Anthropology, University of Cambridge, Cambridge, United Kingdom; University of Oxford, United Kingdom

## Abstract

Ten thousand years before Neolithic farmers settled in permanent villages, hunter-gatherer groups of the Epipalaeolithic period (c. 22–11,600 cal BP) inhabited much of southwest Asia. The latest Epipalaeolithic phase (Natufian) is well-known for the appearance of stone-built houses, complex site organization, a sedentary lifestyle and social complexity—precursors for a Neolithic way of life. In contrast, pre-Natufian sites are much less well known and generally considered as campsites for small groups of seasonally-mobile hunter-gatherers. Work at the Early and Middle Epipalaeolithic aggregation site of Kharaneh IV in eastern Jordan highlights that some of these earlier sites were large aggregation base camps not unlike those of the Natufian and contributes to ongoing debates on their duration of occupation. Here we discuss the excavation of two 20,000-year-old hut structures at Kharaneh IV that pre-date the renowned stone houses of the Natufian. Exceptionally dense and extensive occupational deposits exhibit repeated habitation over prolonged periods, and contain structural remains associated with exotic and potentially symbolic caches of objects (shell, red ochre, and burnt horn cores) that indicate substantial settlement of the site pre-dating the Natufian and outside of the Natufian homeland as currently understood.

## Introduction

Archaeologists have conventionally associated the origins of stone-built architecture with the Late Epipalaeolithic (Natufian) c. 14,500 years ago, and suggest that they represent the first semi-sedentary settlements, marking a critical threshold in human evolution [1]–[3]. Yet, hut structures that suggest repeated and prolonged occupation are acknowledged at several earlier sites and appear as early as 23,000 cal BP at the site of Ohalo II on the shore of the Sea of Galilee [4], [5]. These oval, brushwood structures, recognized archaeologically as dark-stained and organic-rich sediments, have provided evidence for the construction and re-use of a series of successive floors, as well as a division of internal hut spaces for specific activities, such as flint knapping and plant processing [6], [7]. Analysis of the relationships of these structures to each other and other site features, as well as their construction materials and organic and inorganic ‘furniture’ demonstrates their intricate design and regular maintenance. In 2010 excavations at Kharaneh IV, one of the largest Late Pleistocene sites in southwest Asia, revealed additional evidence for Early Epipalaeolithic hut structures. These structures, and their associated features, are significant for several reasons: a) their age, b) ecological setting, and c) evidence for repeated occupation and potentially symbolic behaviors, such as caching. Along with Ohalo II and other Early Epipalaeolithic structures discussed below, they are among the earliest and best preserved dwellings yet found in the region. These dwellings provide new insights into the nature of settlement in the Azraq Basin 20,000 years ago and help to chart the development of early architecture in southwest Asia. They also reinforce a great time depth for the development and flourishing of architecture, prolonged site occupation, and emergent village life prior to the Late Epipalaeolithic Natufian.

## Results

### The Site of Kharaneh IV

**Figure 1:**
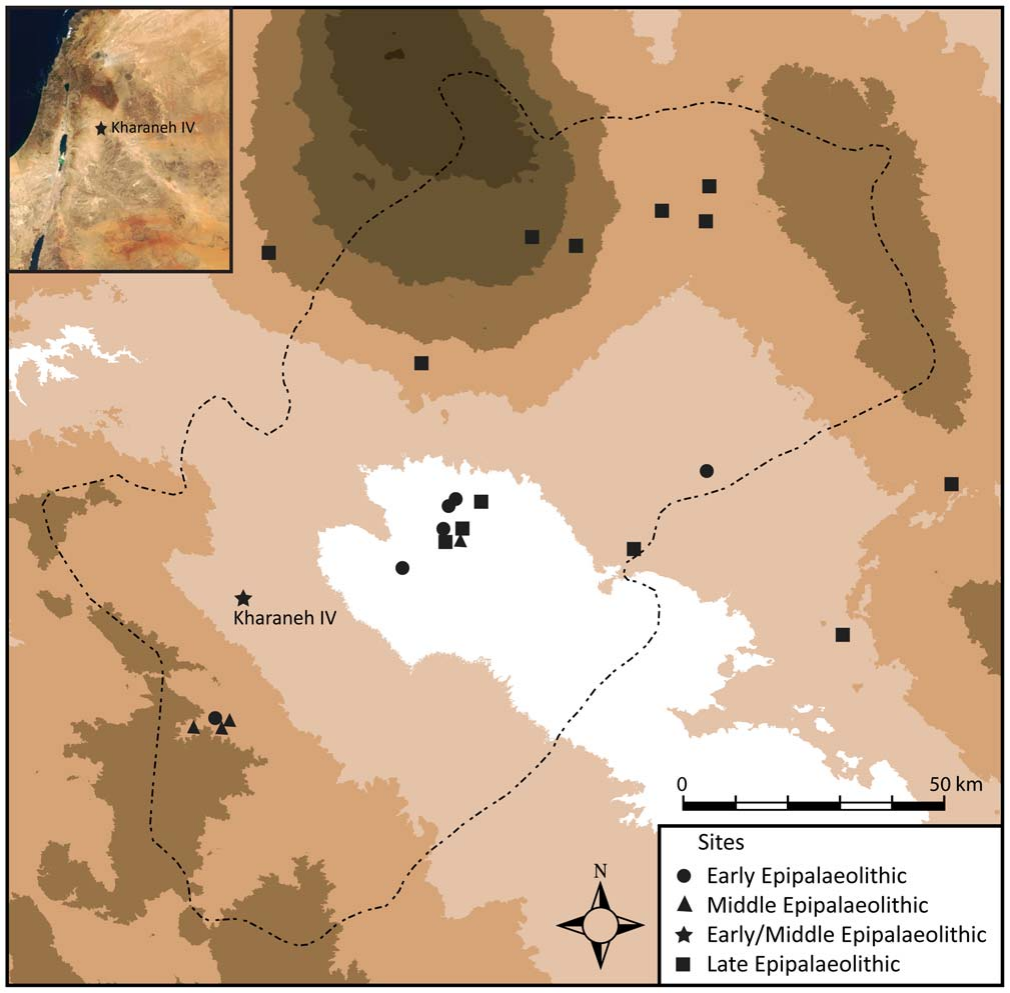
Map of the Azraq Basin showing the location of Kharaneh IV in relation to other Epipalaeolithic sites in the Azraq Basin.

**Figure 2:**
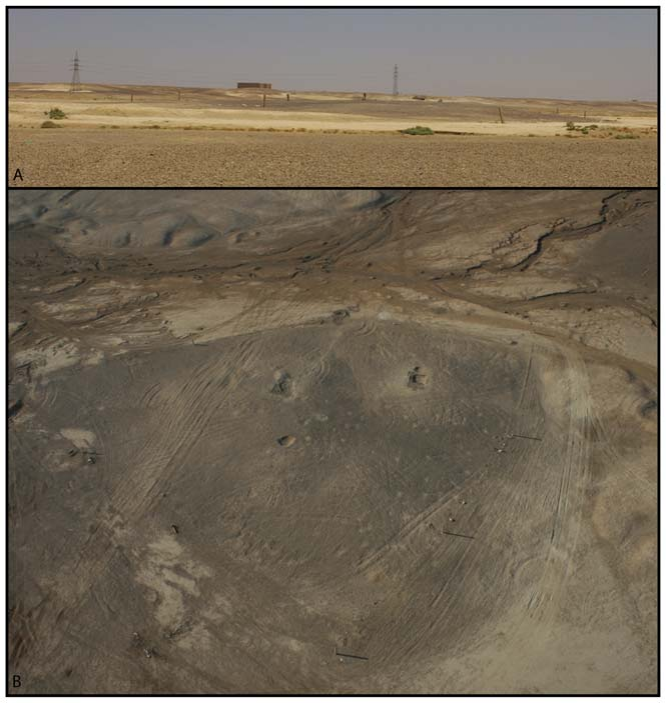
The Early and Middle Epipalaeolithic site of Kharaneh IV. A) A view of the site looking northwards towards Qasr Kharaneh in the background showing the prehistoric mound as it rises above the wadi terrace. B) An aerial view of the site just after excavations in 2008 (photo courtesy of I. Ruben).

Kharaneh IV is situated 70 km east of Jordan's capital Amman at the western edge of the Azraq Basin, a 12,500 km^2^ drainage basin around the Azraq Oasis. Until the 1980s the oasis was a rich and extensive wetland area centered within a semi-arid steppe and desert landscape. The region has long been recognized for its dense concentration of Epipalaeolithic sites [8]–[12] (**Figure 1**). Kharaneh IV covers an area of c. 21,000 m^2^, making it the largest known Late Pleistocene site in the region. Rising almost 2 meters above the surrounding landscape, it is easily identifiable by its staggering concentration of stone tool debris and animal bones (**Figure 2**). Kharaneh IV was first investigated by M. Muheisen in the 1980's who's work provided a preliminary glimpse into the site's stratigraphy and main features, including rare human remains from the Early Epipalaeolithic [13]. The discovery of a number of Late Pleistocene sites in eastern Jordan, some of which are similar in size and density to Kharaneh IV [8], emphasize recent acknowledgment that areas such the Azraq Basin may have formed settlement refugia during harsher periods of the LGM [14]–[16]. Renewed Late Pleistocene research since 2005 by the Epipalaeolithic Foragers of Azraq Project (EFAP) has begun to revisit this important area through new excavation at Kharaneh IV and Ayn Qasiyya, accompanied by a program of intensive palaeoenvironmental research [17]–[25]. Geoarchaeological work on- and off-site provides evidence for a well-watered and well-vegetated Last Glacial Maximum (LGM) habitat in Wadi Kharaneh that highlights the complexity of palaeoenvironmental and palaeoclimatic reconstructions for this and other parts of the Near East region during the LGM e.g. [26], [27], the implications of which are discussed below.

Since 2008 three seasons of excavation at Kharaneh IV have documented approximately one thousand years of repeated and sequential occupation. Thirteen Accelerator Mass Spectrometry dates obtained from the principle excavation areas allow us to confidently date the occupation of the site to between 19.9 and 18.6 ka cal BP (68% confidence level, INTCal 09). Excavations have been carried out in two main areas (Areas A and B) in which prolonged and intense phases of occupation were documented with no evidence for hiatuses in deposition [20]. The uninterrupted sequence is characterized by very dense archaeological deposits (up to 23,000 pieces of chipped stone/m^3^ and similar frequencies of fauna) throughout all occupational horizons. This depth and density of material suggests that people occupied this now-arid area on a protracted basis and gathered at the site regularly to produce such extensive artifact densities over such a large area and depth. While acknowledging that such qualitative terms as prolonged, continuous, or regular occupation are extremely difficult to quantify and discussions regarding the definition of sedentism continue, e.g., [2], [28]–[31], we endeavor to contribute to ongoing debates on the duration of occupation of large aggregation sites with the findings from these structures and continued analyses of artifact assemblages and site-formation processes. As an aggregation site, repeated occupation led to the formation of a complex stratigraphy. Unlike other contemporary sites in the region, Kharaneh IV spans several Epipalaeolithic phases and has rich assemblages of stone tools, worked bone objects, red ochre, and marine shell beads. The preservation of botanical remains, especially charcoal, is excellent. Extensive flotation of all excavated sediments has allowed us to recover a large amount of charcoal from most contexts.

**Figure 3:**
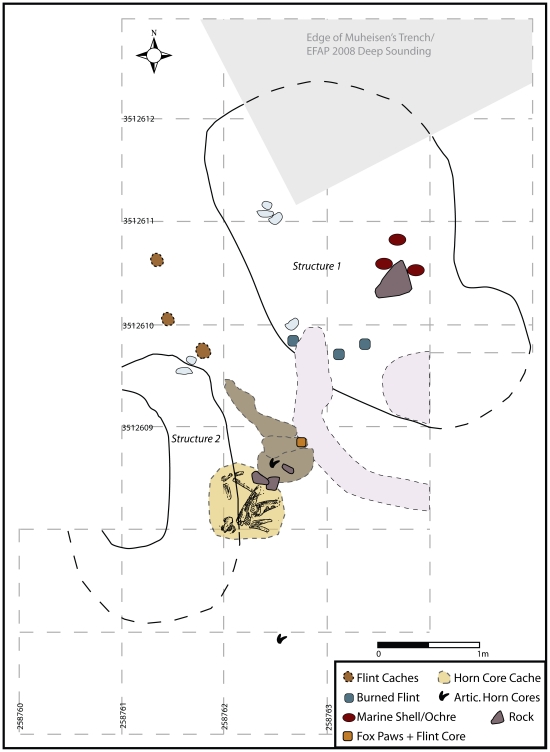
Plan drawing of Structures 1 and 2 showing the dimensions of each hut and their position relative to each other. The light grey shaded area in the top of the plan shows the location of Muheisen's original trench cutting slightly into the corner of Structure 1. Thus, this portion of the edge of Structure 1 is hypothesized from the dimensions of the remainder of the structure. The light pinkish shading shows an area of disturbance cut into a portion of Structure 1's southern boundary.

In Area B our work focuses on a combination of horizontal excavation and vertical exposure of the complete stratigraphic profile of the site's deepest part. The sequence of deposits offers a fine-grained record of the formation of the site. Thin (2–3 cm) and compacted occupation surfaces alternate with thicker (10–15 cm) midden deposits characterized by very dense concentrations of chipped stone and faunal remains. Occupational deposits in Area B reach a depth of 1.35 m below the surface, below which is archaeologically sterile clay representing the deposits of an ancient lake. Charcoal taken directly from the exposed sections here has provided a continuous sequence of radiocarbon dates for the Early Epipalaeolithic spanning from c. 19.9 to 18.9 ka cal BP (68% confidence level, INTCal 09). The lithic industry of Area B is dominated by the production of narrow and gracile bladelets and non-geometric microliths traditionally referred to as belonging to the Kebaran industry. Muheisen reported several large pit features and two human burials in Area B [13], [32]. Our excavations have revealed further pit features, compacted surfaces, numerous hearths, associated middens, and ash dumps. Discussed in detail here, our most recent excavations in 2010 uncovered new evidence for one, possibly two, hut structures dated to 19,400 cal BP (**Figure 3**).

### The Structures

**Figure 4:**
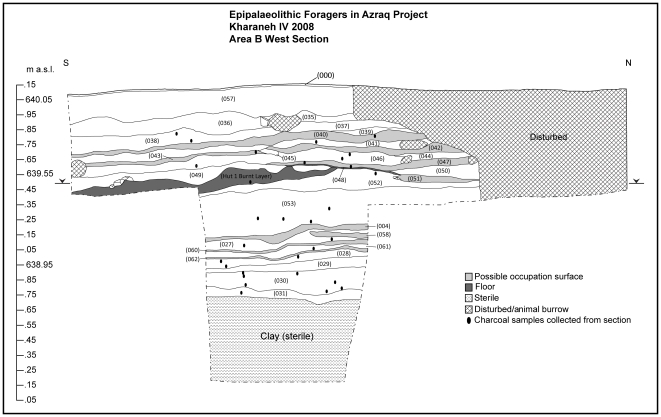
West and South section drawings in Area B showing the stratigraphic relationships between Structure 1 and its surrounding deposits. This section was exposed before the horizontal exposure of hut structure 1 by Muheisen's original sounding in this area and cleaned and drawn by EFAP in 2008.

Structure 1 was exposed approximately 60 cm below modern surface in Area B. It is oval in plan, measuring 3.2 m by 2.2 m and consists of several distinct layers. The structure was placed into a shallow depression dug into the preceding occupation deposits. A thin (2–3 cm), compact, dark reddish-brown clayey deposit represents the former floor of the structure. It is overlain by an organic-rich, black layer (c. 5 cm thick) containing abundant charcoal fragments (**Figure 4**) that represent the residue of *in situ* burning. Burning also reddened the structure's former floor, making its deposits notably different in color and texture from the sediments surrounding the dwelling.

**Figure 5:**
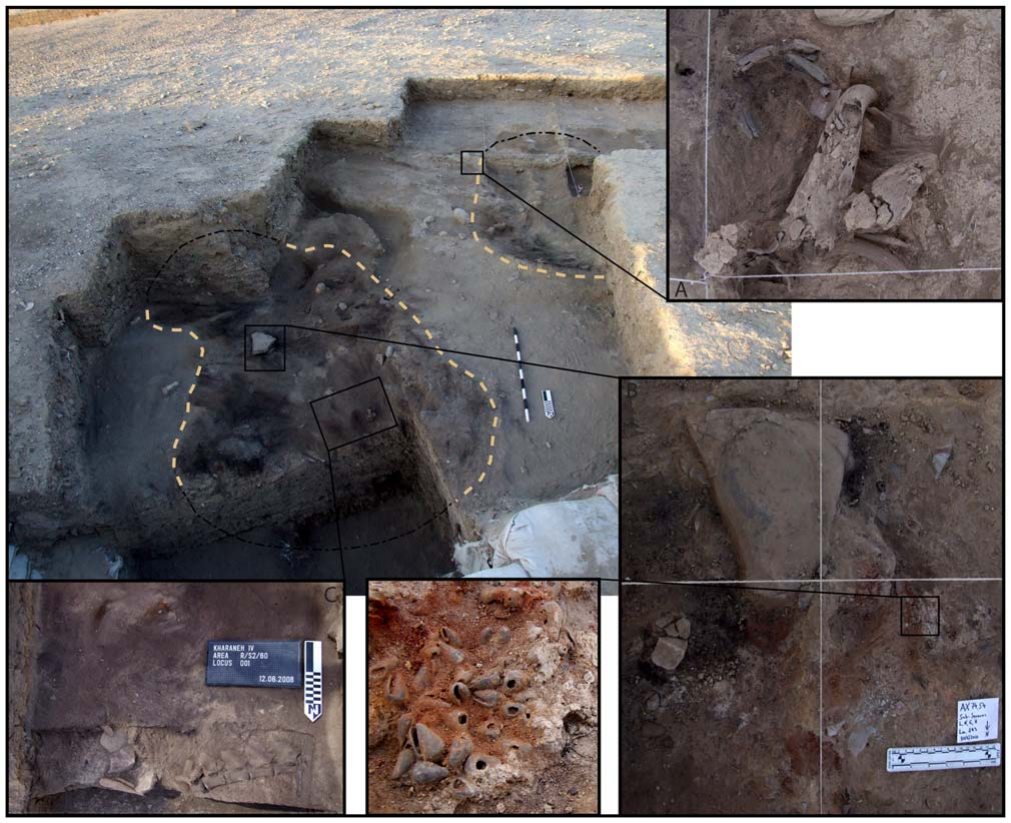
The structures at Kharaneh IV. Two Early Epipalaeolithic structures at Kharaneh IV, showing close-ups of features associated with the structures, including (A) a cache of burned gazelle and aurochsen horn cores at the edge of Structure 2, (B) a large stone associated with three caches of red ochre and pierced marine shells, and (C) articulated *Bos primigenius* lumbar vertebrae and ground stone fragments in the hut foundations.

Sitting on top of the structure's floor and covered by the black burnt layer were two fragments of groundstone, a large flat stone, red ochre, and five articulated wild aurochs lumbar vertebrae (**Figure 5C**). Also nearby, just slightly east of the structures centre, were three distinct concentrations of pierced marine shells, each accompanied by a large (c. 10×5 cm) chunk of red ochre, sitting on top of the burnt layer around a large, flat stone (**Figure 5B**). The entire structure was covered in a brownish-orange, coarse sand largely devoid of artifacts. The three caches of ochre and marine shell produced over 1000 pierced shells, including species from both the Mediterranean and Red Seas, imported to the site over distances of 130 km and 270 km, respectively. The almost sterile, orange sand covering the shell caches has only been found in association with the hut structures (see below). It does not occur in any other archaeological context, nor does it appear naturally in the immediate vicinity of the site, indicating it would have to have been brought in, perhaps to cover the dwellings and their associated features. In addition, large stones are not found naturally in any on-site deposits, so their presence in the hut structure suggests intentional placement.

Muheisen first suggested a possible hut floor in Area B (his Area R/S2/60) in the 1980's when his trench cut into the corner of a dark brown layer (Couche V) beneath which he recorded two human burials [33]. EFAP reopened excavations in Area B partly to fully document these and other associated features. Radiocarbon samples obtained from EFAP's deep sounding in Area B, where the hut floor was first recognized, provide a solid chronological context for Structure 1. Two charcoal samples identified as *Chenopodiaceae* sp. and an indeterminate *Dicot* sp. were radiocarbon dated using Accelerator Mass Spectrometry (OxA 22273: 15,890±90 and OxA 22274: 15770±80, respectively). These samples came from immediately above and immediately below Structure 1's floor and date the structure to between 19.2 and 18.8 ka cal BP (68% confidence, IntCal 09). These dates fit into a stratigraphically coherent sequence of twelve newly obtained dates, which contains no major outliers. We will discuss this sequence in more detail elsewhere.

By analogy with the hut structures from Ohalo II, we interpret the thin, burnt, charcoal-rich layer on top of the structure's floor as the remnants of the hut's former superstructure, which burned and collapsed onto the floor. Further examination of the macro- and micro-botanical remains taken from this layer is currently underway, but the types of charcoal documented in other contexts (see above) suggest it highly likely that this was probably constructed of locally-available vegetation. Shrub and tree charcoal from *Chenopodiaceae* sp. and tamarisk have been identified from associated contexts in Area B (S. Colledge, pers. comm.).

The three concentrations of ochre and marine shells are particularly intriguing. Together these produced as many pierced marine shells as found in the entirety of the remaining excavation areas. Pierced holes strongly suggest that these were pendants of some kind, either worn as necklaces, headdresses, sewn onto clothing or attached to other artifacts [23]. Their import to the Azraq Basin over considerable distances suggests that they may have been considered valuable, potentially as symbolically important objects. Their concentration, orientation around the stone and association with lumps of ochre suggests that they were intentionally placed on top of the burnt remnants of the hut's superstructure. If this were the case, the shells and ochre may represent a post-destruction or post-abandonment offering, and it is possible that the burning of the hut was deliberate. It is intriguing that Muheisen reported the location of the two human burials as being situated beneath the layer of the hut floor (Couche V) [13]. Both of the burials are adult males, one with moderate osteoarthritis. This individual was relatively complete and buried with two large stones over his head and another two over his legs. A photograph of this burial shows one large stone and a gazelle horn core adjacent to his skull. The second individual was reported as a partial burial alongside the first. Unfortunately, the published reports and the remaining excavation records do not allow precise reconstruction of the stratigraphic relationship between hut and human remains. However, as we discuss below, a second hut structure (Structure 2) is also associated with human body parts. It is tempting to link the deliberate placing of the shell and ochre caches and the hut burning with the interment of two human bodies beneath. However, only further excavation and discovery of further human remains at the site will allow us to fully test this hypothesis.

Although only partially excavated, Structure 2 has a similar stratigraphic profile to Structure 1. It consists of a shallow, semi-circular depression dug into existing cultural deposits. Filling the depression is compact, light-colored clay, overlain by several overlapping dark-colored, organic-rich layers that appear to represent several episodes of hut floor re-use. This structure does not show evidence of burning like Structure 1. Notably, this hut also has sterile orange-brown sand in its uppermost levels. The complete dimensions of this structure remain unknown until it is fully excavated; however, it appears similar in size to Structure 1. Although no shell/ochre caches have been found in Structure 2, a cache of gazelle and aurochsen horn cores was found on the edge of the structure (**Figure 5A**). Overlying the hut were midden-like deposits rich in animal bone and chipped stone debris. Dug into this sediment was a pit discernable by a notably higher concentration of semi-articulated animal bones and a single isolated human tibia (right, adult). Isolated human body parts are not uncommon at early Epipalaeolithic sites, and may indicate either the disturbance of burials by later activities, non-interment burial practices (e.g., exposure), or secondary burial practices [24], [34], [35]. It is nevertheless intriguing to note the association of both structures with human remains. We will return to this point later on in our discussion.

**Figure 6:**
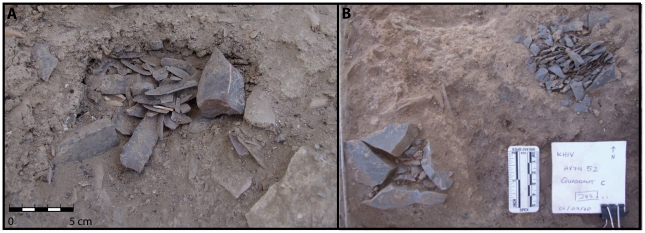
Close-up photograph of A) one of the two chipped stone caches located between Structures 1 and 2. Note the addition of a bone point to this lithic cache. B) *In situ* burned and fractured flint associated with Structure 1.

The distance between these two huts is less than 2 m, and the space in between also contains several interesting features (**Figures 3** **,** **5** **,** **6** **,** **7**). There are two distinct chipped stone concentrations or caches, each of which contains at least one core and several narrow, gracile bladelets and associated knapping debris (chips, core trimming flakes and blades) (**Figure 6A**). One of these caches also contained a bone point. There are also three large chunks of flint that have been extensively burned and are thermally fractured (**Figure 6B**). They are found immediately outside the southern boundary of Structure 1 and are partially covered by sterile orange sand, suggesting they were burned and fractured *in situ* during burning.

**Figure 7:**
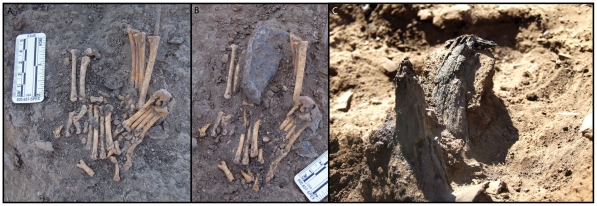
Close-up photographs of A–B) four articulated fox paws surrounding a worked flint bladelet core (B), probably representing the remains and contents of a fox pelt pouch and C) burnt gazelle horn cores still attached to skull at base, standing upright, adjacent to Structure 2.

In addition to these features there was also a hearth that contained a fragment of a grinding slab placed on end along the southern edge of the hearth. Immediately to the north of the hearth (south of Structure 1) was a complete tortoise shell, a large rounded stone, and four articulated fox paws surrounding a flint core that are likely the remains of a pelt pouch (**Figure 7A–B**). To the south and west of the hearth were two sets of gazelle horn cores still attached to the skull at base, burnt and placed upright in the general area around the hearth (**Figure 7C**).

The depositional context and stratigraphy in Area B, as well as the features between them, suggests these huts *may* have been in use at the same time; however, they experienced different life histories. Structure 1 seems to have burned down and been buried with marine shell and ochre. The evidence for abandonment of Structure 2 is less clear and suggested only by the presence of a pit, filled densely with animal bones, and the isolated human tibia, overlying it.

## Discussion

### Epipalaeolithic Architecture

The two huts from Area B are not the only evidence for structures at Kharaneh IV, although they are certainly the clearest discrete features. Area A is characterized by a lithic industry with strong Geometric Kebaran affinity, called Phase D by Muheisen [36], but somewhat distinct from contemporary assemblages documented elsewhere. It consists of trapezes and rectangles, which show a remarkable degree of variation in shape, backing and distal and proximal modification. Seven AMS measurements from Area A date this industry to between 18.6 and 18.8 ka cal BP, although the lithic industry is largely Middle Epipalaeolithic (Geometric Kebaran) in character. Despite its early date, recent reevaluations of Epipalaeolithic radiocarbon data suggests great overlap between the Early and Middle Epipalaeolithic, the implications of which are beyond the scope of this paper [37]. Features associated with this industry in Area A include several overlapping compacted earthen surfaces that are associated with a number of hearths ringed by small post-holes. These surfaces do not appear to be confined within any larger structure and may instead represent outdoor activity areas for food processing – a rarely documented feature in the Middle Epipalaeolithic. Partially-articulated gazelle carcasses and horn cores, as well as the remains of other large game, point towards intensive food processing activities taking place on these surfaces, perhaps including the butchery and preservation of gazelle meat. For example, the post-holes around the hearths may represent drying racks for meat collected from the large numbers of gazelle carcasses.

The discovery of two hut structures at Kharaneh IV, when put together with existing data from Ohalo II and other sites, allows us to place their construction, use and, in the case of Kharaneh IV, possible intentional destruction, within the wider picture of the architectural development in the region. Indeed, huts are not new to the Early Epipalaeolithic and beyond Kharaneh IV and Ohalo II dwellings have been found at a number of other sites, including as Ein Gev I, Nahal Hadera V and Azariq XIII [38]. Yet, durable architecture is really only associated with the Late Epipalaeolithic Natufian and includes stone-built circular structures found at large and dense sites (sometimes also called base camps). Archaeologists have tended to contrast the flimsy, ephemeral, short-term dwellings of the Early and Middle Epipalaeolithic with the more durable, long-lived and solidly-built constructions of the (Early) Natufian [3]. This is further exemplified by reference to earlier Epipalaeolithic structures as ‘huts’ and later Natufian and early Neolithic structures as ‘houses/homes’ (see also discussion in [31]). However, that supposedly more ‘solid’ constructions do not imply more permanent occupation *or* long-term use has not gone unnoticed by researchers [3], [28], [29]. The apparent contrast between earlier Epipalaeolithic and Natufian structures is further highlighted by an increasing emphasis on the non-domestic, ritual use of structures during the Natufian and the Pre-Pottery Neolithic A, and lack of evidence for (but acknowledgement of the possibility of) these ‘special’ uses in earlier phases [3], [38], [39].

New discoveries from Kharaneh IV add to a steadily growing corpus of evidence for Early and Middle Epipalaeolithic dwellings. For example, at the nearby Early Epipalaeolithic site of Jilat 6, small excavations revealed an artificially pigmented ochre surface interpreted as the possible floor of a structure [8]. The two dwelling structures in Area B further document that these are not uncommon features of Early Epipalaeolithic sites. Kharaneh IV is unparalleled in size and artifact density for the entire Epipalaeolithic, Natufian included, and, thus, leads us to question whether we should think of sites such as Kharaneh IV or Ohalo II within existing models of Epipalaeolithic mobility as repeatedly revisited locales within a highly mobile hunting and gathering settlement strategy [40]. There is no reason to assume that stone-built architecture is necessarily more durable than the structures described in this paper or the Ohalo II huts, especially since the walls of Natufian houses are rarely over 2–3 courses in height and may have also had organic superstructures. It is also worth noting that at one of the best-studied Natufian sites in southwest Asia, Tell Abu Hureyra, where year-round occupation and cereal cultivation are said to have first appeared, stone buildings are apparently absent (albeit the Natufian deposits are comparatively small exposures) and with pit and post-hole dwellings present instead [41]. We argue here that Kharaneh IV's size, density, and the presence of structures in both Early and Middle Epipalaeolithic occupations, illustrates that the site was occupied over multiple seasons and sometimes involved the repeated gathering of considerable numbers of people, perhaps as part of customary economic or social events [23], [25]. The Kharaneh IV dwellings therefore shed important insights into occupation of the Azraq Basin and contribute greatly to our bigger picture understanding of Late Pleistocene settlement patterns, the onset of sedentism, and the origins of architecture in southwest Asia.

Several scholars have stressed the symbolic importance of early houses as homes and centers of both ritual and family life when Neolithic villages appeared widely across southwest Asia [3], [38], [39], [42]–[45]. Furthermore, the symbolic associations between houses and ritual/symbolic practices are fairly clearly demonstrated in the Natufian. For example, “In addition to major changes in the size and scope of architectural features during the course of the Natufian, the evidence clearly indicates that these encompassed not only profane aspects but were also imbued with intense symbolic correlates.” [38] Slabs of limestone incised with geometric patterns at Wadi al-Hammeh 27 or the repeated placement of caches of colored pebbles on the floor of structures at Ain Mallaha (Eynan), as well as the interment of the dead beneath house floors (at Eynan and Hayonim Cave), have all been provided as examples for these symbolic associations. Despite acknowledgement of the probable symbolic aspects of Early and Middle Epipalaeolithic dwellings and related structures, they have rarely been addressed in any detail – a factor likely related to the rarity of such structures to-date. Now, sites such as Kharaneh IV, Ohalo II, and others, allow us to examine changes in the size and scope of early architecture, and the relationships between ritual and mundane, within a longer time frame. While there is general agreement that developments within the Natufian and Neolithic had their origins earlier in the Epipalaeolithic (e.g., [15], [16]), the data presented here provide further and tangible demonstrations of this trend.

The evidence presented here for the placement of ochre and pierced marine shell caches, possibly intentional burning of Structure 1, human remains, and the associated caches between the two dwellings at Kharaneh IV reinforce that Natufian and early Neolithic instances of symbolism associated with domestic structures are not new or unique. Previous distinctions between mobile Early/Middle Epipalaeolithic groups and later Natufians used the argument that sedentism (presumed on the basis of large, dense sites, non-portable ground stone, and stone buildings) and social complexity (presumed largely from portable art and ornamentation, cemeteries, and caches), and its associated symbolic behavior, arose with the onset of the Natufian period [42], [46], [47]. An increasing corpus of data from earlier Epipalaeolithic sites demonstrate both a longer time depth for these behaviors *and* that searching for the ‘first’ huts, houses, sedentary sites or ritual behaviors, as we currently understand them, may be a futile enterprise. The Kharaneh IV structures and the associations between their destruction, ochre-pierced marine shell caches and, possibly, human remains would seem to indicate that our distinctions between domestic and symbolic are still unclear and may hinder a more nuanced understanding of the changes and transformations in human behavior throughout the Epipalaeolithic and Neolithic in southwest Asia.

### The Azraq Landscape

One of EFAP's primary archaeological questions regarding occupation of Kharaneh IV, now in one of the driest areas of the region, is why people chose this location and repeatedly occupied the site at least a thousand years. Off-site and on-site geomorphological work demonstrates that the Late Pleistocene landscape was very different from that of today. Instead of a marginal environment, the conditions prevailing during the LGM appear to have facilitated a moister local climate than today with the likelihood of year-round rivers and bodies of standing water outside of the oasis, in a landscape that supported a diversity of plant and animal species and extensive and intensive human occupation [19]. Large amounts of charcoal from the archaeological deposits support a reconstruction of grasslands and trees (e.g., chenopods, tamarisk, wild pistachio), with availability of a diversity of plant species for food, fuel, and construction. In essence, rather than being a marginal desert environment removed geographically from the social and cultural transformations occurring to the west, the Azraq Basin and, especially, Kharaneh IV was an attractive locale for repeated and prolonged occupation, long before the appearance of Natufian base camps.

To-date, Epipalaeolithic sites with hut structures have only been documented in the western and southern (northern Negev) parts of the southern Levant – the former of these areas sometimes referred to as the Mediterranean core (see [3], [38] for a detailed overview of the evidence). Their presence here has suggested that this area was more amenable to long-term occupation and, thus, the foci of cultural developments [38], [46]. We suggest that the structures at Kharaneh IV, and repeated occupation of the site suggested by the Early and Middle Epipalaeolithic deposits, are evidence that long-term occupation or aggregation sites were not just isolated to the presumably rich Mediterranean zone. They are also found in the higher altitude (>600 m asl) and ecologically-distinct Azraq Basin in eastern Jordan. The nature and types of occupation we see at Kharaneh IV helps shed light onto how we understand the development of sedentism and architecture in the Late Epipalaeolithic Natufian (see also [38]). We see now that the scope of these transitions is larger than previously thought and, indeed, the movement of and interaction between Early and Middle Epipalaeolithic groups are quite widespread and intensive.

The evidence for hut structures in the Early Epipalaeolithic, at Kharaneh IV and elsewhere, demonstrates that these were features of repeatedly-occupied, probably multi-season, sites. Furthermore, these structures pre-date Natufian stone architecture and were not confined to the supposedly lush Mediterranean zone of the Levant. Although detailed analyses of the huts, their sediments, and associated artifacts are ongoing they provide evidence that the Ohalo II structures are not isolated instances. Perhaps more importantly for this discussion, the Kharaneh IV huts show that the local environment could support long-term settlement which, together with palaeoenvironmental evidence for well-watered environments near the site [17], [19], suggests that this was far from a ‘marginal’ environment. The 20,000 year-old huts at Kharaneh IV provide direct new evidence for the complex nature of settlement and long-distance social relations at Jordan's elusive aggregation sites and demonstrates that dense, large-scale settlements appeared early in the Epipalaeolithic record in the now-desert areas of eastern Jordan that were once productive grasslands with significant water sources.

## Materials and Methods

The field work under discussion here was carried out during three excavation seasons between 2008 and 2010. All necessary permits were obtained from the Department of Antiquities of Jordan for the described field studies.

A main focus of EFAP's work is to reconstruct the Late Pleistocene palaeoenvironment to contextualize hunter-gatherer behavior; therefore, field work necessarily integrates archaeological, biological, and geomorphological datasets. We have begun to reconstruct a picture of site occupation, subsistence behaviors, and some of the social aspects of repeated occupation. To provide a more holistic reconstruction of landscape and land use EFAP is undertaking a rigorous program of sampling for archaeobotanical remains and conduct flotation for 100% of all subsurface deposits. The excellent preservation conditions in this now-desert environment enable the collection of botanical remains that are not often preserved on archaeological sites of any period. The result is an extensive record of habitat change and plant use, as well as the ability to radiocarbon date all contexts.

The site is excavated in a grid system 200 m (east-west) by 140 m (north-south) in size, covering an area slightly larger than the surface spread of artefacts to allow for future exploration (if required) off-site to the north and east. The grid is labelled with an alphanumeric system beginning in the southwestern-most square metre of the gridded universe, and has a letter designation running along the Y-axis (north-south) and a number designation running along the X-axis. Each 1 m^2^ is identified by its SW corner alphanumeric designation such that the southwestern-most 1 m^2^ of the site is labelled Square A1. All other 1 m^2^ excavation units follow this same system.

Excavations have focussed in two main areas, Area A and Area B, but 1×1 m test trenches have been placed in several locations across the surface of the site and a 9×1 m geological trench traced the transition from on-site occupational deposits to the modern wadi margins. A total of 104 m^2^ have been excavated to-date. In both Area A and Area B, excavation was conducted on our basic site grid of 1×1 m excavation squares. However, sometimes these squares were subdivided into 50×50 cm quadrants or 25×25 cm sub-quadrants where dictated by the stratigraphic context (e.g., the presence of features such as hearths, pits, floors). Structure 1 was excavated in 25×25 cm sub-quadrants. Vertical subdivisions of natural and cultural stratigraphic layers into 5–10 cm thick spits were used in order to record as much spatial information as possible. For the uppermost disturbed levels we screened 100% of excavated deposits on-site through 4 mm and 2 mm mesh. However, this proved to be very time-consuming on-site given the density of flint and bone from surface deposits. Given the potential for preservation of plant and animal remains that could greatly inform us on season and duration of occupation at the site, as well as on-site activities and subsistence practices, 100% of deposits were collected for flotation in the field lab. Flotation was conducted daily in the field lab along with sorting of the 4 mm heavy fraction from flotation. In addition, samples were systematically collected for micro-artefacts, micro-fauna, micromorphology, and soil analyses that aid in reconstructing activities at the site and general site-formation processes.

## References

[1] Bar-Yosef O (1998). The Natufian Culture in the Levant: Threshold to the Origins of Agriculture.. Evolutionary Anthropology.

[2] Belfer-Cohen A, Bar-Yosef O, Kuijt I (2000). Early Sedentism in the Near East: A Bumpy Ride to Village Life.. Life in Neolithic Farming Communities: Social Organization, Identity, and Differentiation.

[3] Goring-Morris AN, Belfer-Cohen A, Bocquet-Appel J-P, Bar-Yosef O (2008). A Roof Over One's Head: Developments in Near Eastern Residential Architecture Across the Epipalaeolithic-Neolithic Transition.. The Neolithic Demographic Transition and its Consequences.

[4] Nadel D (2002). Ohalo II. A 23,000-year-Old Fisher-Hunter-Gatherer's Camp on the Shore of the Sea of Galilee.

[5] Nadel D, Belitzky S, Boaretto E, Carmi I, Heinemeier J, Bruins HJ, Carmi I, Boaretto E (2001). New Dates From Submerged Late Pleistocene Sediments in the Southern Sea of Galilee, Israel.. Radiocarbon: Near East Chronology, Archaeology and Environment.

[6] Nadel D, Otte M (1995). The Organization of Space in a Fisher-Hunter-Gatherers Camp at Ohalo II, Israel.. Nature et Culture: Colloques de Liège.

[7] Nadel D, Werker E (1999). The oldest ever brush hut plant remains from Ohalo II, Jordan Valley, Israel (19 000 BP).. Antiquity.

[8] Garrard A, Byrd B (1992). New dimensions to the epipalaeolithic of the Wadi el-Jilat in Central Jordan.. Paléorient.

[9] Garrard A, Baird D, Byrd B, Bar-Yosef O, Kra R (1994). The chronological basis and significance of the Late Palaeolithic and Neolithic sequence in the Azraq Basin, Jordan.. Late Quaternary Chronology and palaeoclimates of the Eastern Mediterranean.

[10] Betts AVG (1998). The Harra and the Hamad: Excavations and Surveys in Eastern Jordan, Volume 1.

[11] Copeland L, Hours F (1989). The Hammer on the Rock: Studies in the Early Palaeolithic of Azraq, Jordan Part I.

[12] Rollefson G, Quintero L, Wilke P (2001). Azraq Wetlands Survey 2000, Preliminary Report.. Annual of Department of Antiquities of Jordan.

[13] Muheisen M, Garrard A, Gebel H (1988). The Epipalaeolithic phases of Kharaneh IV.. The Prehistory of Jordan: The State of Research in 1986.

[14] Goring-Morris AN, Levy TE (1995). Complex hunter-gatherers at the end of the Paleolithic (20,000-10,000 BP).. The Archaeology of the Holy Land.

[15] Goring-Morris AN, Hovers E, Belfer-Cohen A, Shea J, Lieberman D (2009). The Dynamics of Pleistocene and Early Holocene Settlement Patterns and Human Adaptations in the Levant: An Overview.. Transitions in Prehistory: Essays in Honor of Ofer Bar-Yosef.

[16] Goring-Morris N, Belfer-Cohen A (1998). The articulation of cultural processes and the late quaternary environmental changes in Cisjordan.. Paléorient.

[17] Jones M, Richter T, Allcock S, Maher L, Martin L (2010). Late Pleistocene environments of occupation in the Azraq Basin, Jordan. 7th International Conference on the Archaeology of the Ancient Near East.

[18] Maher L, Warren G, Finlayson B (2010). People and their places at the end of the Pleistocene: evaluating perspectives on physical and cultural landscape change.. Landscapes in Transition: Understanding hunter-gatherer and farming landscapes on the early Holocene of Europe and the Levant.

[19] Jones M, Richter T (2011). Palaeoclimatic and archaeological implications of Pleistocene and Holocene environments in Azraq, Jordan.. Quaternary Research.

[20] Maher L, Richter T, Stock J, Jones M, Khraysheh F, Rollefson G (In press). Preliminary Results from Recent Excavations at the Epipalaeolithic Site of Kharaneh IV.. Jordan's Prehistory: Past and Future Research.

[21] Richter R, Healey E, Campbell S, Maeda O (2011). Nebekian, Qalkhan and Kebaran: variability, classification and interaction. New insights from the Azraq Oasis.. The State of the Stone Terminologies, Continuities and Contexts in Near Eastern Lithics.

[22] Richter T, Alcock S, Jones M, Maher L, Martin L (2010). New light on Final Pleistocene settlement diversity in the Azraq Basin: some preliminary results from Ayn Qasiyah.. Paléorient.

[23] Richter T, Garrard A, Allcock S, Maher L (2011). Interaction Before Agriculture: Exchanging Material and Shared Knowledge in the Final Pleistocene Levant.. Cambridge Archaeological Journal.

[24] Richter T, Stock JT, Maher L, Hebron C (2010). An Early Epipalaeolithic Sitting Burial from the Azraq Oasis, Jordan.. Antiquity.

[25] Martin L, Edwards Y, Garrard A (2010). Hunting Practices at an Eastern Jordanian Epipalaeolithic Aggregation Site: The Case of Kharaneh IV.. Levant.

[26] Enzel Y, Amit R, Dayan U, Crouvi O, Kahana R (2008). The climatic and physiographic controls of the eastern Mediterranean over the late Pleistocene climates in the southern Levant and its neighboring deserts.. Global and Planetary Change.

[27] Robinson SA, Black S, Sellwood BW, Valdes PJ (2006). A review of palaeoclimates and palaeoenvironments in the Levant and Eastern Mediterranean from 25,000 to 5000 years BP: setting the environmental background for the evolution of human civilisation.. Quaternary Science Reviews.

[28] Boyd B (2006). On sedentism in the Later Epipalaeolithic (Natufian) Levant.. World Prehistory.

[29] Edwards P (1989). Problems of recognizing earliest sedentism: the Natufian example.. Journal of Mediterranean Archaeology.

[30] Hardy-Smith T, Edwards PC (2004). The Garbage Crisis in prehistory: artefact discard patterns at the Early Natufian site of Wadi Hammeh 27 and the origins of household refuse disposal strategies.. Journal of Anthropological Archaeology.

[31] Finlayson B, Mithen SJ, Najjar M, Smith S, Maricevi D (2011). Architecture, sedentism, and social complexity at Pre-Pottery Neolithic A WF16, Southern Jordan.. Proceedings of the National Academy of Sciences.

[32] Rolston SL (1982). Two prehistoric burials from Qasr Kharaneh.. Annual of the Department of Antiquities of Jordan.

[33] Muheisen M (1983). La Préhistoire en Jordanie. Recherches sur l'Epipaléolithique. L'Example du Gisement de Kharaneh IV.

[34] Maher LA, Stock JT, Finney S, Heywood JJN, Miracle P (2011). A Unique Human-Fox Burial from a Pre-Natufian Cemetery in the Southern Levant (Jordan).. PLoS ONE.

[35] Nadel D (1994). Levantine Upper Palaeolithic - Early Epipalaeolithic Burial Customs: Ohalo II as a Case Study.. Paléorient.

[36] Muheisen M, Wada H (1995). An Analysis of the Microliths at Kharaneh IV Phase D, Square A20/37.. Paléorient.

[37] Maher L, Banning EB, Chazan M (2011). Oasis or Mirage? Assessing the Role of Abrupt Climate Change in the Prehistory of the Southern Levant.. Cambridge Archaeological Journal.

[38] Goring-Morris N, Belfer-Cohen A, Vasil'ev SA, Kozlowski J (2003). Structures and Dwellings in the Upper and Epi-Palaeolithic (c.42-10k BP) Levant: Profane and Symbolic Uses.. Perceived Landscapes and Built Environments: The cultural geography of Late Paleolithic Eurasia.

[39] Grosman L, Munro N (2007). The sacred and the mundane: domestic activities at a Late Natufian burial site in the Levant.. Before Farming: the archaeology and anthropology of hunter-gatherers.

[40] Maher L, Richter T, Stock J (2012). The Early and Middle Epipalaeolithic of the Southern Levant: Developments and Transitions to Social Complexity.. Evolutionary Anthropology.

[41] Moore AM, Hillman GC, Legge AJ (2000). Village on the Euphrates: From Foraging to Farming at Abu Hureyra.

[42] Verhoeven M (2004). Beyond Boundaries: Nature, Culture, and a Holistic Approach to Domestication in the Levant.. Journal of World Prehistory.

[43] Watkins T (1992). The Beginning of the Neolithic: searching for meaning in material culture change.. Paléorient.

[44] Watkins T, Córdoba JM, Molist M, Pérez MC, Rubio I, Martínez S (2006). Natural environment versus cultural environment: The implications of creating a built environment..

[45] Watkins T, Finlayson B, Warren G (2010). Changing People, Changing Environments: How Hunter-Gatherers Became Communities that Changed the World.. Landscapes in Transition.

[46] Byrd B (2005). Reassessing the Emergence of Village Life in the Near East.. Journal of Archaeological Research.

[47] Gilead I (1988). The Upper Palaeolithic to Epi-Palaeolithic Transition in the Levant.. Paléorient.

